# Naturally-acquired humoral immune responses against the N- and C-termini of the *Plasmodium vivax *MSP1 protein in endemic regions of Brazil and Papua New Guinea using a multiplex assay

**DOI:** 10.1186/1475-2875-9-29

**Published:** 2010-01-21

**Authors:** Carmen Fernandez-Becerra, Sergi Sanz, Marina Brucet, Danielle I Stanisic, Fabiana P Alves, Erney P Camargo, Pedro L Alonso, Ivo Mueller, Hernando A del Portillo

**Affiliations:** 1 Barcelona Centre for International Health Research (CRESIB), Hospital Clinic/IDIBAPS, Universitat de Barcelona, Rosello 132, 08036 Barcelona, Spain; 2 Papua New Guinea Institute of Medical Research, Madang MP511, Papua New Guinea; 3 Infection and Immunity Division, Walter and Eliza Hall Institute of Medical Research, 1G Royal Parade, Parkville, Australia; 4 Departamento de Parasitologia, Instituto de Ciencias Biomedicas, Universidade de São Paulo, São Paulo, Brazil; 5 Institució Catalana de Recerca I Estudis Avançats (ICREA), Barcelona, Spain

## Abstract

**Background:**

Progress towards the development of a malaria vaccine against *Plasmodium vivax*, the most widely distributed human malaria parasite, will require a better understanding of the immune responses that confer clinical protection to patients in regions where malaria is endemic.

**Methods:**

Glutathione *S*-transferase (GST) and GST-fusion proteins representing the N- terminus of the merozoite surface protein 1 of *P. vivax*, PvMSP1-N, and the C-terminus, PvMSP1-C, were covalently coupled to BioPlex carboxylated beads. Recombinant proteins and coupled beads were used, respectively, in ELISA and Bioplex assays using immune sera of *P. vivax *patients from Brazil and PNG to determine IgG and subclass responses. Concordances between the two methods in the seropositivity responses were evaluated using the Kappa statistic and the Spearman's rank correlation.

**Results:**

The results using this methodology were compared with the classical microtitre enzyme-linked immnosorbent assay (ELISA), showing that the assay was sensitive, reproducible and had good concordance with ELISA; yet, further research into different statistical analyses seems desirable before claiming conclusive results exclusively based on multiplex assays. As expected, results demonstrated that PvMSP1 was immunogenic in natural infections of patients from different endemic regions of Brazil and Papua New Guinea (PNG), and that age correlated only with antibodies against the C-terminus part of the molecule. Furthermore, the IgG subclass profiles were different in these endemic regions having IgG3 predominantly recognizing PvMSP1 in Brazil and IgG1 predominantly recognizing PvMSP1 in PNG.

**Conclusions:**

This study validates the use of the multiplex assay to measure naturally-acquired IgG antibodies against the merozoite surface protein 1 of *P. vivax*.

## Background

*Plasmodium vivax *is the most widely distributed human malaria parasite and responsible for 100-300 million clinical cases each year including severe disease and death [1]. Studies on naturally acquired immune responses against different *P. vivax* antigens, including, among others, merozoite surface antigens, Duffy-binding protein, circumsporozoite surface protein, apical membrane 1, have demonstrated their immunogenicity in natural infections [2-6]. In addition, some of these studies have also demonstrated differences in acquisitiuon of IgG subclasses against particular antigens. The association of clinical protection with specific antigens of *P. vivax*, however, has been reported in only two prospective cohort longitudinal studies [7,8]. In the first, clinical protection was associated with IgG3 antibodies against the N-terminus of the merozoite surface protein 1 (PvMSP1) in residents of the Brazilian Amazon region of Portuchuelo [7]. In the second, clinical protection was reported in children from Papua New Guinea [8]. This latter study used a functional assay on naturally-acquired binding inhibitory antibodies against the Duffy-binding protein region II (PvDBPII) to demonstrate the association of these antibodies with protection against *P. vivax *infection.

Antibodies in these studies were measured using the ELISA assay [9], a technique requiring high amounts of coating-antigens and immune sera when large numbers of samples must be screened. To overcome these limitations, suspension array technologies with high-throughput capacity to simultaneously analyse several proteins with minimal amount of immune sera have been developed [10]. Recently, these methodologies have been reported to measure antibodies to multiple malaria vaccine candidate antigens [11], to measure simultaneously antibody recognition of twenty eight *Plasmodium falciparum *erythrocyte membrane protein 1 (PfEMP1) domains [12], and to develop a high throughput functional assay to study binding of ICAM1 to the 3D7 strain PfEMP1 repertoire [13].

The aim of this study was to measure naturally acquired IgG anti-PvMSP1 antibodies in human patients infected with *P. vivax *from different endemic regions of Brazil and Papua New Guinea through implementation and validation of the BioPlex suspension array system.

## Methods

### Human samples and study areas

Human plasma samples were obtained from different endemic areas of Brazil and PNG and one control group from a non-endemic region. One group comprised 87 adults who participated of a cross-sectional survey at riverine communities from Rio Machado in the Brazilian Amazon [14]. The other group consisted of 122 children from Madang and Maprik in Papua New Guinea who presented to local Health Centers (with a median age of 30.5 months and age range from three to 72 months). One hundred human plasma samples were used to analyse the concordance between the BioPlex assay and ELISA. The remaining samples were used to measure IgG isotypes. Control subjects consisted of 16 healthy adult volunteers living in the city of Barcelona (Spain) that never have been exposed to malaria or visited malaria endemic regions. These studies received the ethical approval of Local Institutional Reviewing Boards.

### Recombinant antigens

Glutathione *S*-transferase (GST) and GST-fusion proteins representing the N- terminus, PvMSP1-N, and the C-terminus, PvMSP1-C, have already been described [2,15]. GST and GST-fusion proteins were purified on glutathione-Sepharose 4B (GE healthcare), and protein concentration was determined by Bradford assay (Bio-Rad).

### Covalent coupling of recombinant proteins to beads

BioPlex carboxylated beads (Bio-Rad) were covalently coated with the different recombinant proteins following the manufacturer's instructions (BioPlex Amine Coupling Kit). Briefly, activated beads (1.25 × 10^6 ^beads/ml) were resuspended in 100 μl of PBS and 1 μg of each recombinant protein used per coupling reaction. Incubation under rotation was done at 4°C overnight and coupled beads were washed with 500 μl of PBS pH 7.4. After re-suspending coupling beads in 250 μl of blocking buffer and further incubation under rotation at room temperature for 30 min, beads were washed with 500 μl of storage buffer and centrifuged for six minutes at 14,000 × g. Pellets were ressuspended into 125 μl of the same buffer and stored at 4°C protected from light until use.

### Analysis of coupled beads on the BioPlex system

Coupled beads were analysed as described in Cham *et al *[12] with modifications. Briefly, aliquots of 50 μl, corresponding to 5,000 coated beads were used for each assay. Frozen plasma samples were thawed at room temperature, diluted 1:50 in assay buffer and 50 μl aliquots added to the beads (final plasma dilution 1:100). Aliquots of 50 μl of Biotinylated human IgG antibody (Sigma) diluted 1:7,500 and of phycoerythrin conjugated streptavidin diluted to 2 μg/ml were used in subsequent incubations. Beads were re-suspended in 125 μl of assay buffer (BioRad) and analysed on the BioPlex100 system and results were expressed as median fluorescent intensity (MFI). The BioPlex assay to detect IgG subclasses was performed as described previously using specific biotinylated human IgG isotypes (Sigma) diluted 1:5,000 in assay buffer. Specifically, monoclonal anti-human IgG1 clone 8c/6-39, monoclonal anti-human IgG2 clone HP-6014, monoclonal anti-human IgG3 clone HP-6050 and monoclonal anti-human IgG4 clone HP-6025 were used.

### Enzyme-linked immunosorbent assay (ELISA)

Human IgG antibodies against GST and GST-PvMSP1 tags were detected by ELISA as described [2]. Plates were coated with 100 ng/well of each protein. The OD_492 _values to each recombinant protein were obtained by subtracting the OD492 values of the same serum to GST alone. Cut-off points were set at three standard deviations above the mean OD_492 _of sera from 16 individuals, unexposed to malaria, from the city of Barcelona.

### Statistical analysis

Non-parametric test (Fisher exact and Wilcoxon Rank Sum) were carried out to test differences with response variable when appropriate. Univariate and multivariate logistic regression were adjusted to analyse the relationship on the seropositivity of PvMSP1-C or PvMSP1-N measured by ELISA or BioPlex techniques. Correlation among variables was evaluated with Spearman's rank correlation.

Concordances between the two methods in the seropositivity responses were evaluated using the Kappa statistic. Good agreement was defined as kappa-value > 0.6, according to [16]. All the tests were performed using a two-sided significance level of 0.05. The analysis was performed using STATA version 9.2 (StataCorp., College Station, TX, USA).

## Results

### Concordance between the BioPlex assay and ELISA

**Figure 1:**
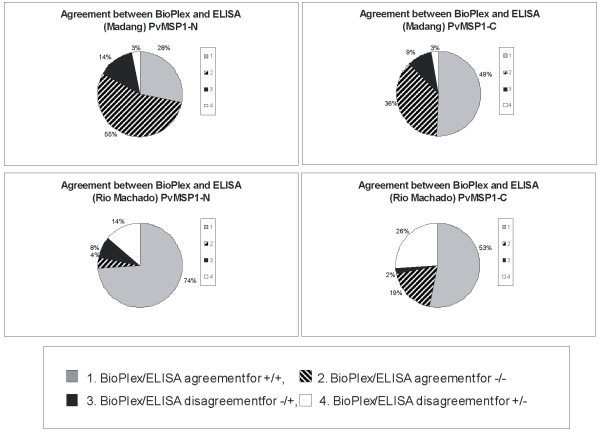
**Diagram representing the concordance between BioPlex and ELISA assays**.

Three recombinant proteins corresponding to GST and GST-fused to the N- and C-termini of PvMSP1 were used for this analysis. Plasma samples from 87 adults from Brazil as well as from 100 children from PNG were tested at 1:100 dilutions to measure total IgG antibodies. Concordance between the BioPlex and ELISA assays was evaluated using the Kappa statistics. Kappa values presented good agreement for the samples from Madang and for both recombinant proteins, PvMSP1-N (Kappa = 0.638 and total agreement = 83%) and PvMSP1-C (Kappa = 0.758 and total agreement = 88%) (Figure 1). The concordance of both techniques using sera from Rio Machado was lower for both molecules, PvMSP1-N (Kappa = 0.163 and total agreement = 78%) and PvMSP1-C (Kappa = 0.366 and total agreement = 72%) (Figure 1).

**Figure 2:**
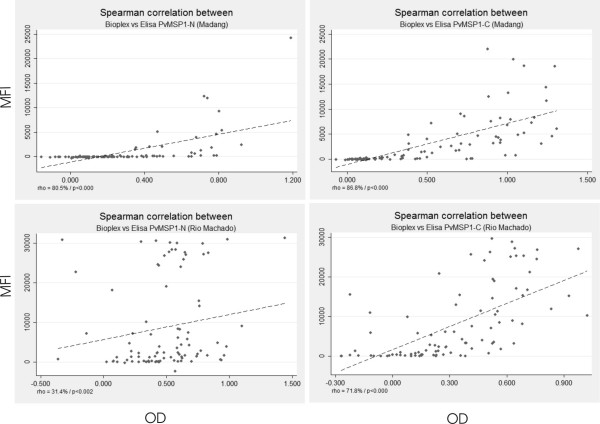
**Correlation between the results from BioPlex and ELISA using the Spearman's rank correlation test**. Rho: Spearman's rank correlation coefficient, MFI (BioPlex): median fluorescent intensity and OD (ELISA): Optical density.

Concordance between BioPlex and ELISA assay was also evaluated with Spearman's rank correlation (Figure 2). The Spearman's rank correlation coefficients (Rho) for the samples from Madang were respectively 80.5% and 86.8% for PvMSP1-N and PvMSP1-C. The Spearman's rank correlation coefficients (Rho) for the samples from Rio Machado was lower for PvMSP1-N with a Rho = 34.4%, and for PvMSP1-C with a Rho = 71.8%. In spite of these different values, correlations were significant (p = 0.005) for all comparisons.

### Detection of total IgG antibodies to PvMSP1 by BioPlex assay and association with risk factors

Percentage of individuals from Madang and Rio Machado containing naturally-acquired IgG antibodies against PvMSP1 are presented in Table 1. IgG antibodies to PvMSP1-N and PvMSP1-C were detected, respectively, in 31 (31%) and 52 (52%) (Madang) and 77 (89%) and 70 (80%) (Rio Machado). When groups were divided into symptomatic, asymptomatic and non-infected individuals (defined as subjects with no symptoms, negative microscopy and/or negative PCR), 15/50 (30%) and 8/9 (89%) of symptomatic patients from Madang and Rio Machado contained, respectively, anti-PvMSP1-N antibodies whereas 28/50 (56%) from Madang and 9/9 (100%) from Rio Machado reacted against PvMSP1-C. Furthermore, 7/20 (35%) and 38/42 (90%) of asymptomatic patients from Madang and Rio Machado recognized, respectively, PvMSP1-N whereas 12/20 (60%) and 30/42 (71%) reacted against PvMSP1-C. Of importance, a high number of non-infected patients, 9/30 (30%) from Madang and 31/36 (86%) from Rio Machado contained specific anti-PvMSP1-N antibodies and 12/30 (40%) from PNG and 31/36 (86%) from Rio Machado antibodies against PvMSP1-C.

**Table 1:** Percentage of individuals positive for IgG antibodies to PvMSP1 N- and C-termini in samples from PNG (Madang) and Brazil (Rio Machado) obtained by BioPlex assay.

	Samples from Madang n = 100		Samples from Rio Machado n = 87	
	
Rec. protein		Classification		Classification
PvMSP1-N	31 (31%)	S 15/50 (30%)	77 (89%)	S 8/9 (89%)
		AS 7/20 (35%)		AS 38/42 (90%)
		N.I. 9/30 (30%)		N.I. 31/36 (86%)
				
PvMSP-C	52 (52%)	S 28/50 (56%)	70 (80%)	S 9/9 (100%)
		AS 12/20 (60%)		AS 30/42 (71%)
		N.I. 12/30 (40%)		N.I 31/36 (86%)

S (symptomatic patients), AS (asymptomatic patients), N.I. (not infected).

Univariate and multivariate logistic regressions analysis showed that, excepting for age, no other variable was significantly associated with anti-PvMSP1 antibodies (Table 2). Interestingly, age was only associated with antibodies against the C-terminus but not the N-terminus in both areas. Moreover, in Rio Machado the association was observed in older people (positive individuals mean age = 32 years, negative individuals mean age = 23 years), whereas in PNG was observed in younger children (positive individuals mean age = 27 months; negative individuals mean age = 37 months).

**Table 2:** Results of the multilevel logistic regression model showing different variables and their association with IgG antibodies to PvMSP1 N- and C- termini in samples from Brazil (Rio Machado) and Papua New Guinea (Madang).

	Samples from Rio Machado			Samples from Madang		
	
Variable	OR	95%CI	p-value	OR	95%CI	p-value
**PvMSP N-terminus**						
						
Classification						
Non infected	1			1	(1.00-1.08)	
Symptomatic	1.29	(0.13-12.66)		1.00	(0.37-2.69)	
Asymptomatic	1.53	(0.38-6.20)	0.835	1.26	(0.38-4.20)	0.911
Sex						
Female	1					
Male	0.26	(0.05-1.27)	0.096	n.d		
Age (years)						
Unit increment	1.04	(0.99-1.10)	0.113	1.01	(0.98-1.03)	0.607
						
**PvMSP1C-terminus**						
						
Classification						
Non infected	1			1		
Asymptomatic	0.40	(0.13-1.28)	1.24	1.91	(0.76-4.79)	
Symptomatic				2.25	(0.71-7.14)	0.283
Sex						
Female	1					
Male	1.65	(0.60-4.55)	0.336	n.d		
Age						
Unit increment	1.04	(1.00-1.08)	0.046	0.97	(0.95-0.99)	0.013

### Prevalence of IgG subclasses

**Figure 3:**
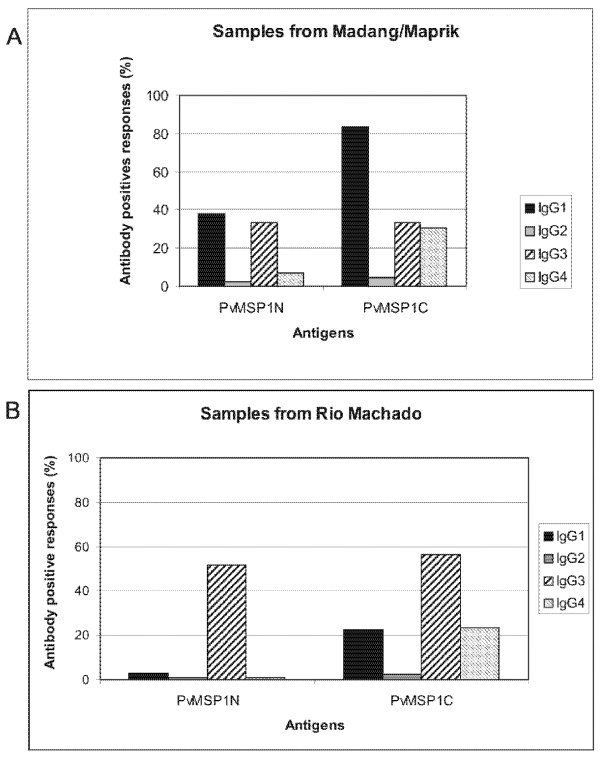
**IgG subclass profiles against PvMSP1-N and PvMSP1-C**. A. Percentage of IgG subclass responses in Madang/Maprik (PNG). B. Percentage of IgG subclass responses in Rio Machado (Brazil).

To analyse the IgG subclass antibody responses to PvMSP1, 42 plasma samples from Brazil and 42 from PNG were selected. Samples from Brazil included asymptomatic patients (29/42) as previous results had already investigated their IgG isotype profiles [7]. Remaining samples from Brazil and PNG were randomly chosen. Results demonstrated that children from PNG contained predominantly IgG1 antibodies to PvMSP1-C (82%) and lower percentages of IgG3 and IgG4 (close to 40%) to both N- and C-termini of PvMSP1 (Figure 3A), whereas adults from Brazil contained predominantly IgG3 antibodies to the N- and C-termini of PvMSP1 (close to 60%) (Figure 3B). None of the variables studied was significantly associated with any one particular IgG isotype.

## Discussion

The use of multiplex assays has not been reported for *P. vivax *antigens and, therefore, a comparison of the BioPlex assay values with those obtained by ELISA was initially made to determine the concordance and correlation of these techniques. To do so, two different statistics, the Kappa statistics and the Spearman's rank correlation test, were used. Kappa Statistics has been proposed as the most appropriate method when the same parameter with two similar techniques is compared [16]. The coefficient correlation tests, such as Spearman's rank correlation or Pearson correlation, has been used to validate multiplex assays in *P. falciparum *and other diseases [11,12,17]. Using Kappa statistics, a good agreement, with significant values, was obtained for both recombinant proteins with the samples from PNG. In contrast, even if the agreement of the results obtained from Brazil was above 70%, Kappa values were not significant. Most of the discrepancy, however, was the result of samples slightly positive by BioPlex and negative by ELISA. In addition, the Spearman's rank correlation test demonstrated significant correlations for all comparisons (Figure 2). As this correlation has been used to validate the use of multiplex assays in *P. falciparum *and other diseases, the data from Brazil remain meaningful; yet, it also argues that further research into different statistical analyses is desirable before claiming conclusive results exclusively based on multiplex assays.

Naturally-acquired IgG responses against PvMSP1 have demonstrated that a large proportion of individuals expose to *P. vivax *contain IgG antibodies against different regions of the molecule [15,18,19]. The results obtained with the BioPlex assay with subjects from Brazilian adults and PNG children confirm these findings even though a lower overall response to PvMSP1 was observed in PNG children (Table 1). Interestingly, non-infected individuals (no symptoms, negative microscopy and/or negative PCR) from both regions contained antibodies against PvMSP1. This data indicate that anti-PvMSP1 antibodies are long-lived and/or that these individuals presented a sub-patent parasitaemia, not detected by microscopy or PCR, representing undetected carriers. Both explanations have supporting evidence as anti-PvMSP1 antibodies have been reported to last up-to 30 years [20] and direct mosquito feeding of patients with sub-patent vivax infections have successfully transmitted *P. vivax *to mosquitoes [21]. Determining their frequencies will have implications for malaria control programmes in these regions.

Studies on naturally acquired immunity in *P. vivax *in PNG have shown that it is acquired in children below nine years of age [22], while acquired immunity in Brazil is only reached later, when closer to 15-20 years old in native populations [7] and also in migrant adult working populations [18]. Interestingly, age was significantly associated with the response to PvMSP1-C and not to PvMSP1-N in both regions; yet, in PNG this association was mainly observed in younger children (positive individuals mean age = 27 months, negative individuals mean age = 37 months) whereas in Brazil was observed in older populations (positive individuals mean age = 32 years, negative individuals mean age = 23 years). Further prospective studies with larger sample sizes are required to determine the role, if any, of PvMSP1 in age-dependent acquisition of immunity.

This is the first study that uses the BioPlex system to measure IgG subclasses in malaria. To validate its use, sera from asymptomatic patients from Brazil were chosen as previous studies had demonstrated that these patients contained predominantly IgG3 antibodies against PvMSP1-N [7]. As expected, IgG3 anti-PvMSP1-N antibodies were the predominant isotype in asymptomatic patients from Rio Machado. Remaining IgG profiles were very much in agreement with previous studies using ELISA in which IgG1 and IgG3 cytophilic isotypes are the predominant isotypes against PvMSP1 with low or undetectable levels of IgG2 and IgG4 [19,23]. Interestingly, IgG isotype profiles of PNG and Brazil were different with a predominance of IgG1 antibodies against PvMSP1 in PNG and a predominance of IgG3 antibodies against PvMSP1 in Brazil. Here again, whether this result is due to the difference in the age groups, remains to be determined in larger surveys from these regions including all age-groups.

## Conclusions

Several studies on naturally acquired IgG responses against different *P. vivax *antigens have been described using the ELISA assay. The BioPlex assay described and validated here is the first to measure naturally acquired humoral IgG antibody responses against a *P. vivax *antigen and the first one to study IgG subclasses in malaria. These studies compared the naturally acquired humoral IgG responses against PvMSP1 in different endemic regions of Brazil and PNG. Results demonstrated differences in percentage of responders, differences between the age correlating to responses and differences on isotype profiles. Whether these differences are due to the limited power of this study, which was mainly designed to validate the use of the multiplex assay for PvMSP1, or to real differences in the immunoepidemiology of *P. vivax *in these endemic regions, is presently under investigation. Most relevant, prospective comparative longitudinal cohort studies in endemic different regions using the BioPlex assay can now be envisaged to look for associations against infection and clinical protection having clear implications in antigen discovery and vaccine development for *P. vivax*.

## Competing interests

The authors declare that they have no competing interests.

## Authors' contributions

CFB produced recombinant proteins and implemented the BioPlex assay. MB performed ELISA assays. SS and FA performed the statistical analysis. EPC & FA provided samples from Rio Machado. DS provided samples from PNG. DS, FA, PLA and IM critically read the manuscript. CFB and HAP conceived this study and drafted the manuscript. All authors' read and approved the final manuscript.
